# Allografts as alternative to autografts in primary posterior cruciate ligament reconstruction: a systematic review and meta-analysis

**DOI:** 10.1007/s00167-022-07258-y

**Published:** 2022-11-30

**Authors:** Filippo Migliorini, Andrea Pintore, Francesco Oliva, Jörg Eschweiler, Andreas Bell, Nicola Maffulli

**Affiliations:** 1 Department of Orthopaedic, Trauma, and Reconstructive Surgery, RWTH University Hospital, 52074 Aachen, Germany; 2 Department of Orthopaedic and Trauma Surgery, Eifelklinik St.Brigida, 52152 Simmerath, Germany; 3 Department of Orthopaedics, Surgery and Dentistry, University of Salerno, Via S. Allende, 84081 Baronissi, SA Italy; 4 Barts and the London School of Medicine and Dentistry, Centre for Sports and Exercise Medicine, Queen Mary University of London, Mile End Hospital, 275 Bancroft Road, London, E1 4DG England; 5 School of Pharmacy and Bioengineering, Faculty of Medicine, Keele University, Thornburrow Drive, Stoke on Trent, England

**Keywords:** Knee, Posterior cruciate ligament reconstruction, Allograft, Autograft

## Abstract

**Purpose:**

Following posterior cruciate ligament (PCL) rupture, autografts and allografts are routinely used for its reconstruction. This study investigated the efficacy and safety of allografts for primary PCL reconstruction, comparing them to autografts in terms of patient-reported outcome measures (PROMs), functional tests, and complications.

**Methods:**

This study followed the PRISMA guidelines. PubMed, Web of Science, Google Scholar, Embase, and Scopus were accessed in October 2022. All the clinical studies investigating the outcomes of primary PCL reconstruction using allografts, or comparing the outcomes of allografts versus autografts, were accessed. The outcomes of interests were: instrumental laxity, range of motion (ROM), Telos stress radiography, drawer test, International Knee Documentation Committee (IKDC), Tegner Activity Scale, and the Lysholm Knee Scoring Scale. Data on complications were also recorded.

**Results:**

A total of 445 patients were included. The mean follow-up was 45.2 ± 23.8 months. The mean age of the patients was 30.6 ± 2.2 years. The time span between the injury and surgical intervention was 12.9 ± 10 months. Overall, 28% (125 of 445 patients) were women. Good baseline comparability was found between the two cohorts. No difference was found in terms of Lysholm Score, ROM, Tegner Scale, IKDC, arthrometer laxity, drawer test, and Telos stress radiography. No difference was found in the rates of anterior knee pain and revision.

**Conclusion:**

Allografts can be considered a suitable alternative to autografts for PCL reconstruction.

**Level of evidence:**

III.

## Introduction

The posterior cruciate ligament (PCL) provides primary stabilization for posterior tibial translation over the femur [7, 36, 46, 58]. The PCL is typically injured by a high-energy trauma directed posteriorly to the proximal tibia with the flexed knee, an event which typically occurs in traffic accidents or contact sports practice [37, 55]. Sport-specific PCL injuries incidence ranges from 1 to 4% [3]. The PCL has better healing capability and greater strength than the anterior cruciate ligament [17, 60]. Indeed, PCL tears can be managed conservatively when up to 10 mm of posterior knee translation is present [1]. However, surgical PCL reconstruction may be necessary in patients with greater laxity associated with instability [38, 54]. Both autografts and allografts have been employed for PCL reconstruction [4, 46, 53]. In 2018, Belk et al. [4] performed a systematic review including five studies [2, 27, 31, 52] comparing allografts versus autografts for PCL reconstruction. Since then, further available studies have not yet been considered for systematic reviews [13, 21, 33, 47, 48, 56, 57, 59, 60].

The present study investigated the role of allografts for primary PCL reconstruction, comparing them to autografts in terms of patient-reported outcome measures (PROMs) and functional tests. It was hypothesized that allografts and autografts achieve similar outcome following reconstruction of the PCL.

## Materials and methods

### Search strategy

This systematic review followed the Preferred Reporting Items for Systematic Reviews and Meta-Analyses: the PRISMA checklist [40]. The PICOT algorithm was primarily developed:

- P (Population): PCL tears;
- I (Intervention): PCL reconstruction;
- C (Comparison): allograft versus autograft;
- O (Outcomes): joint instability, PROMs.
- T (Timing): minimum 12 months follow-up.

### Data source and extraction

Two authors (F.M. and A.P.) independently performed the literature search in October 2022. PubMed and Web of Science were accessed. Subsequently, Google Scholar, Embase, and Scopus were accessed to identify further articles. The following keywords were used in combination: knee, posterior cruciate ligament, PCL, tears, injury, surgery, reconstruction, allograft, autograft, instability, BPTB, Achilles, hamstring, tibialis anterior, quadriceps, laxity, patient-reported outcome measures, PROMs, laxity, stability, complication, anterior knee pain, failure, revision. The same authors independently performed the initial screening of the resulting titles. If title and abstract matched, the article full-text was accessed. The bibliographies of the full-text articles were also screened. Disagreements were solved by a third author (N.M.).

### Eligibility criteria

All the clinical studies investigating the outcomes of primary PCL reconstruction using allografts, or comparing the outcomes of allografts versus autografts, were accessed. Given the authors’ language capabilities, articles in English, German, Italian, French, and Spanish were included. Studies of level I–IV of evidence, according to Oxford Centre of Evidence-Based Medicine [23], were considered. Editorials, cohort studies, reviews, technical notes, narrative reviews, expert opinion, and letters were excluded. Animal, biomechanics, and cadaveric studies were also excluded. Articles combining PCL reconstruction with ACL reconstruction were excluded. Articles reporting data on revision settings were not considered. Only articles reporting quantitative data under the outcomes of interest were considered for inclusion.

### Data extraction

Two authors (F.M. and A.P.) independently performed data extraction. Study generalities (author, year, journal, type of study) and patient baseline demographic information (number of samples with related gender and mean age, time span between the injury and the index surgery, length of the follow-up) were collected. The following data were retrieved at last follow-up for both grafts: (1) functional tests: instrumental laxity, range of motion (ROM), Telos stress radiography, drawer test; (2) PROMs: International Knee Documentation Committee (IKDC), Tegner Activity Scale, Lysholm Knee Scoring Scale; (3) complications. The instrumental laxity was evaluated using the arthrometers KT-1000 or KT-2000 (MEDmetric Corp, San Diego, California). Both these devices applied a posterior translation force of 134 N on the tibial plateau over the femur condyles.

### Methodology quality assessment

For the methodological quality assessment, the Coleman Methodology Score (CMS) was used. The CMS is widely used to evaluate the methodological quality of scientific articles for systematic reviews and meta-analyses [12]. This score analyses the included papers under several items, including study size, length of the follow-up, surgical approach, type of study, and the description of diagnosis, surgical technique, and rehabilitation. Additionally, outcome criteria assessment, procedures for assessing outcomes, and the subject selection process are also evaluated. The CMS rates articles with values comprised between 0 (poor) and 100 (excellent). A mean value greater than 60 points is considered satisfactory.

### Statistical analysis

All statistical analyses were performed by the main author (F.M.). All the studies reporting the outcomes of PCL reconstruction using allografts were compared to autografts. Data from autografts were obtained from the included studies which compared between the two grafts. Continuous variables were analysed using the mean difference (MD) effect measure. The Student *t* test was performed, with values of *P* < 0.05 considered statistically significant. The odds ratio (OR) effect measure was used to investigate the rate of complications, with values of the $\chi$^2^ test < 0.05 considered statistically significant. Studies that directly compared allografts versus autografts which also reported measure of data dispersion (standard deviation or confidence interval) were included in the meta-analysis. The meta-analyses were performed using the Review Manager Software 5.3 (The Nordic Cochrane Collaboration, Copenhagen). The inverse variance was adopted for continuous variables, with MD effect measure. Dichotomous data were evaluated through a Mantel–Haenszel analysis, with OR effect measure. The comparisons were performed with a fixed model effect as set up. Heterogeneity was assessed through the Higgins-*I*^2^ test. If *I*^2^ test > 50%, high heterogeneity was detected. In cases of heterogeneity, a random model effect was used. The confidence intervals (CI) were set at 95% in all comparisons. The overall effect was considered statistically significant if *P* < 0.05. The funnel plot of the most commonly reported outcome was performed to assess the risk of publication bias. Egger’s linear regression was performed through the STATA MP Software version 16 (StataCorp, College Station, USA) to assess funnel plot asymmetry, with values of *P* < 0.05 indicating statistically significant asymmetry.

## Results

### Search result

**Fig. 1:**
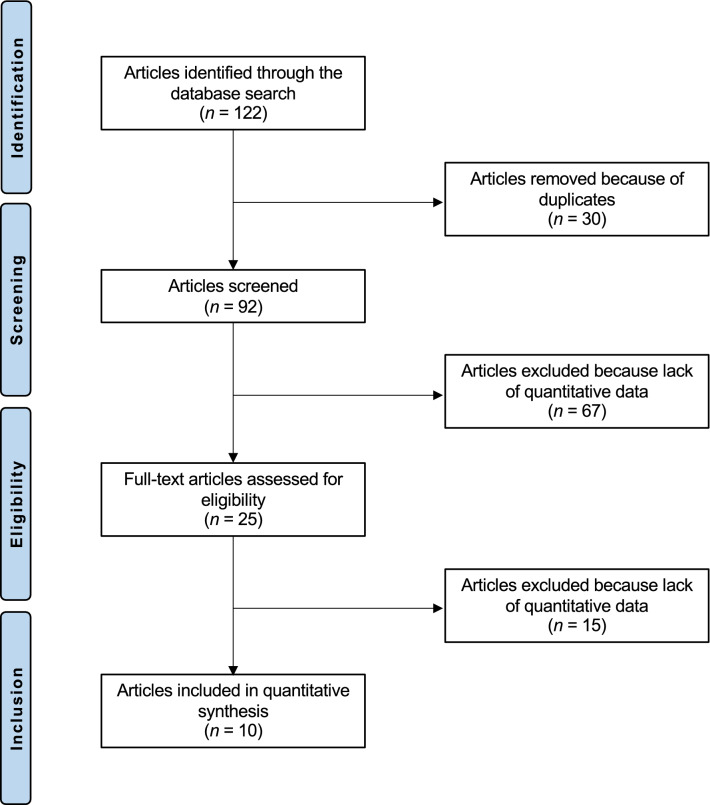
Flow chart of the literature search

The literature search identified 122 articles comparing allografts versus autografts for primary posterior cruciate ligament reconstruction. Of these, 30 duplicates were excluded. An additional 67 articles were excluded because of the following reasons: revision setting (*n* = 4), type of study (*N* = 50), combined with ACL reconstruction (*N* = 7), combined allografts and allografts (*N* = 6). An additional 15 articles were excluded because they lacked quantitative data under the outcomes of interest. This left 10 comparative studies for inclusion: 7 retrospective studies, and three prospective studies (Fig. 1).

### Methodological quality assessment

The CMS identified some limitations and strengths in the present study. The study size and the length of the follow-up were adequate in most of studies. Surgical approach, diagnosis, and rehabilitation protocols were generally well described. Outcome measures and timing of assessment were often well defined, providing moderate reliability. The procedures for assessing outcomes and subject selection were often biased and not satisfactorily described. Concluding, the CMS for the articles was 66 points, attesting the acceptable quality of the methodological quality assessment.

### Risk of publication bias

**Fig. 2:**
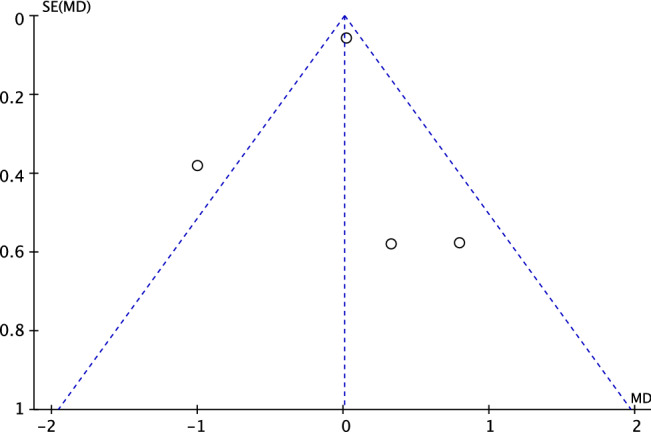
Funnel plot of the most reported outcome (instrumental laxity)

The funnel plot of the most reported outcome was performed (Fig. 2). The graph evidenced minimal asymmetry in the referral point disposition. However, the Egger’s test did not evidence any statistically significant asymmetry (*P* = n.s.). In conclusion, the risk of publication bias was low to moderate.

### Patient demographics

A total of 445 patients were included. The mean follow-up was 45.2 ± 23.8 months. The mean age of the patients was 30.6 ± 2.2 years. The time span between the injury and surgery was 12.9 ± 10 months. Overall, 28% (125 of 445 patients) were women. Good baseline comparability was found between the two cohorts in terms of mean age, length of the follow-up, time span between the injury and the surgical intervention. The demographics of the included studies is shown in Table 1.

**Table 1:** Demographics of the included studies (BPTB: bone–patellar tendon–bone)

Author, year	Journal	Design	CMS	Graft	Treatment	Follow-up (months)	Patients (*n*)	Mean age	Female (*%*)
Ahn et al. 2005 [2]	Arthroscopy	Retrospective	67	Autograft	Hamstring	35.0	18	30.0	16.6
				Allograft	Achilles	27.0	18	31.0	33.3
Cooper et al. 2004 [13]	Am J Sports Med	Prospective	78	Autograft	BPTB	39.4	16	28.0	24.4
				Allograft	BPTB		25		
Hermans et al. 2009 [21]	Am J Sports Med	Retrospective	61	Autograft	BPTB Hamstring	109.2	25	30.8	12.0
				Control group	Hamstring Achilles				
				Allograft	Achilles				
Li et al. 2014 [31]	Knee Surg Sports Traumatol Arthrosc	Retrospective	65	Autograft	Hamstring	27.6	18	31.3	27.7
				Allograft	Tibialis anterior	28.8	19	32.5	36.8
Ma et al. 2019 [59]	Indian J Orthop	Prospective	83	Autograft	Hamstring	28.0	60	33.6	30.0
				Allograft	Achilles	28.0	30	31.5	26.6
Rauck et al. 2018 [47]	Phys Sportsmed	Retrospective	66	Autograft	Quadriceps Hamstring	75.6	8	27.5	43.0
				Autograft	Hamstring		1		
				Allograft	Achilles		6		
Razi et al. 2020 [48]	BMC Muscoloskelet Disord	Retrospective	53	Autograft	Tibialis posterior	37.7	7	25.6	0.0
				Allograft			10		
Sun et al. 2015 [52]	Arch Med Sci	Retrospective	63	Autograft	Hamstring	37.2	36	31.1	25.0
				Allograft	Hamstring	39.6	35	33.4	22.2
Wang et al. 2004 [56]	Injury	Prospective	58	Autograft	Quadriceps	33.0	32	29.0	21.8
					Hamstring				
				Allograft	Achilles	34.0	23	30.0	30.4
					Tibialis anterior				
Wang et al. 2017 [57]	J Int Med Res	Retrospective	70	Autograft	Hamstring	71.6	41	32.0	44.8
				Allograft	Hamstring	71.6	17	32.0	44.8

### Outcomes of interest

No difference was found between allografts and autografts in Lysholm Score, ROM, Tegner Activity Scale, IKDC, arthrometer laxity, drawer test, and Telos stress radiography. These results are shown in greater detail in Table 2.

**Table 2:** Overall results of the two cohorts

Procedures	Autograft	Allograft	MD	*P* value
Lysholm score	86.6 ± 3.2	87.5 ± 3.3	− 0.9	n.s
ROM mean (°)	131.7 ± 5.9	131.7 ± 4.0	0.0	n.s
Tegner activity scale	6.5 ± 1.4	6.4 ± 1.3	0.1	n.s
IKDC	78.7 ± 3.3	77.1 ± 4.1	1.6	n.s
Drawer test	0.7 ± 0.5	0.6 ± 0.4	0.1	n.s
Arthrometer laxity	3.5 ± 0.8	3.5 ± 1.0	0.0	n.s
Telos stress radiography (mm)	9.1 ± 6.7	2.9 ± 0.1	6.2	n.s

### Complications

No difference was found in the rate of anterior knee pain and revision. These results are shown in greater detail in Table 3.

**Table 3:** Complications

Endpoint	Autograft	Allograft	95% CI	OR	*P* value
Anterior knee pain	4/32	0/32	0.53 to 199.01	10.3	n.s
Revision	1/18	0/18	0.01 to 8.73	0.3	n.s

### Meta-analyses

**Fig. 3:**
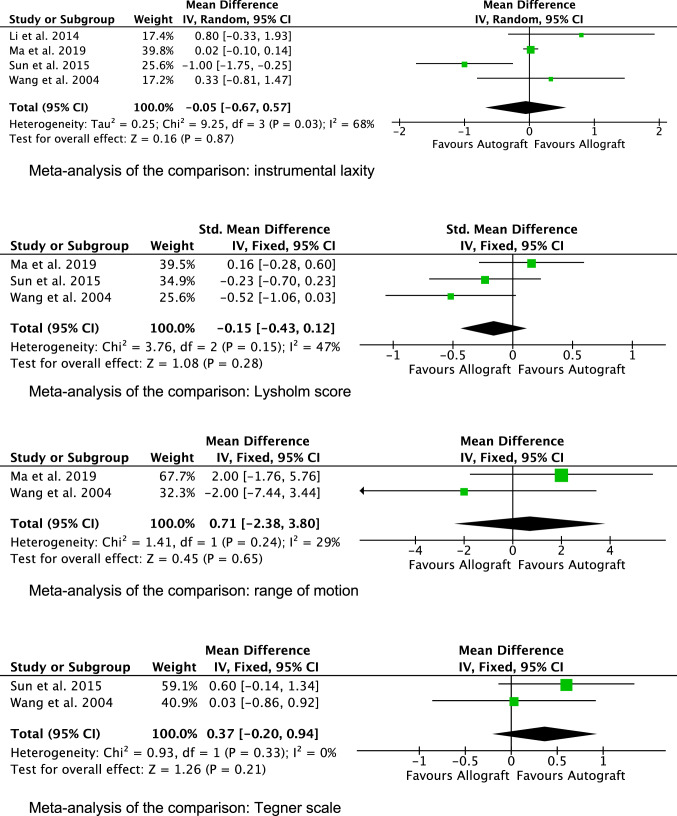
Forest plots of the comparisons included in the meta-analysis

Five studies [2, 31, 52, 56, 59] compared directly allografts versus autografts and were included in the meta-analysis. No difference was found in Lysholm Score, ROM mean, Tegner Activity Scale, and arthrometer laxity. The forest plots of the comparisons included in the meta-analysis are shown in Fig. 3.

## Discussion

The main finding of the present study is that allografts demonstrated similar outcome to autografts for PCL reconstruction. No differences were observed in functional tests (arthrometer laxity, ROM, Telos stress, drawer test), PROMs (IKDC, Lysholm Knee Scoring Scale) and activity levels according to the Tegner Activity Scale. Only one revision over 445 procedures was reported (0.2%). The PCL is the strongest of the knee ligaments, approximately twice as strong as the ACL [26], and revision procedures following its reconstruction are rare. Future clinical studies providing longer follow-up are required to investigate possible difference in graft survivorship.

Semitendinosus, gracilis, bone–patellar tendon–bone (BPTB), and quadriceps autografts are commonly used for PCL reconstruction [5, 8–11, 14–16, 18, 20, 24, 35, 42, 44, 51, 62]. Among allografts, given its length and thickness, the Achilles tendon is commonly employed for PCL reconstruction [31]. Hamstring, tibialis anterior, and BPTB allografts have also been used [29, 39, 43, 50, 61]. Advantages of allografts include a shorter surgical duration, no donor site morbidity, and the choice of desired graft length and thickness. On the other hand, allografts have high costs, potential risk of disease transmission, the possibility of graft versus host reaction, and potential early deterioration deriving from sterilization methods [29, 39, 43, 50, 61]. After implantation, autografts undergo incorporation and remodelling, developing characteristics similar to the native ligament [34, 45]. This process of “ligamentization” is minimal or absent in allografts [30]. A recent systematic review investigated factors influencing the biomechanical properties of allografts [28]. High-dose irradiation for sterilization purposes decreased tensile strength and stiffness compared to low-dose protocols [28]. Further, prolonged freezing impaired the load to failure, ultimate stress and ultimate strain of grafts. Several chemical sterilization measures also negatively affect the biomechanical properties [28]. Surgeons must be aware of the processes that allografts underwent, to better adapt the PCL reconstruction to individual patients.

A recent systematic review including five studies (132 patients) compared allografts versus autografts for PCL reconstruction [4]. They found greater anteroposterior knee laxity in the allograft group with similar Lysholm, IKDC, and Tegner scores, concluding that probably both grafts provide similar outcomes [4]. To date, there is no consensus with regard to the graft choice for primary PCL reconstruction, and the graft source relates to the surgeon preferences. Different autografts have been used for PCL reconstruction [5, 8–11, 14–16, 18, 20, 24, 35, 42, 44, 51, 62]. Setyawan et al. [51] described PCL reconstruction using peroneus longus tendon autograft with good functional outcomes and preservation of ankle function at 2 years follow-up. Recently, Rhatomy et al. [49] compared peroneus longus versus hamstring autografts, reporting excellent postoperative knee functional outcome scores for both groups. The maximum strength of hamstring tendon autograft is comparable to the biomechanical proprieties of patellar tendon autograft [22]. However, some concerns have been reported with the use of BPTB, including anterior knee pain, kneeling pain, risks of patellar fracture and weakening of the extensor mechanism, which acts as a synergist to the PCL [22]. Lin et al. [32] found that both hamstring and BPTB autografts achieved similar good clinical outcome; however, the latter evidenced greater rates of kneeling, squatting, and anterior knee pain [32]. Achilles tendon, tibialis anterior and posterior, BTPB, and hamstring tendons are commonly used as allografts [29, 31, 39, 43, 50, 61]. Achilles tendon allografts are expensive and not always available [6, 41]. Quadriceps allograft could be also considered a good alternative to the Achilles allograft for PCL reconstruction [25]. Achilles and quadriceps allografts for PCL reconstruction demonstrated similar clinical outcomes [25]. In a biomechanical study, Achilles allografts showed more similar biomechanical characteristics of a native PCL compared to quadriceps allografts [19].

The findings of the present study must be interpreted with some limitations. The relatively small number of studies available for inclusion represents an important limitation. Further, 76% (10 of 13 included studies) were retrospective, increasing the risk of selection bias. Moreover, the analyses were conducted irrespective of the source and the strands of the grafts. The current literature lacks prospective analyses with blinding or sample randomization. The eligibility criteria and the procedure protocols were often biased and small between-study differences were evidenced. Allografts must be increased for revision PCL reconstruction, but there is no evidence yet to recommend allografts compared to autograft for primary PCL reconstruction.

## Conclusion

Allografts are an effective and safe alternative to autografts in PCL reconstruction.

## Data Availability

The data underlying this article are available in the article and in its online supplementary material.

## Abbreviations

PCL: Posterior cruciate ligament
PROMs: Patient-reported outcome measures
ROM: Range of motion
IKDC: International Knee Documentation Committee
